# Binding Mode Analysis of Antifouling Compounds Targeting Tyrosinase and Acetylcholinesterase by Saturation Transfer Difference NMR Spectroscopy

**DOI:** 10.1002/cbic.70311

**Published:** 2026-04-20

**Authors:** Ana Sara Gomes, Mariana Andrade, Diana I. S. P. Resende, Sara M. Cravo, Emília Sousa, Marta Correia‐da‐Silva

**Affiliations:** ^1^ CIIMAR/CIMAR LA Centro Interdisciplinar de Investigação Marinha e Ambiental Terminal de Cruzeiros do Porto de Leixões Universidade do Porto Matosinhos Portugal; ^2^ CEMUP Centro de Materiais Universidade do Porto Porto Portugal; ^3^ Faculdade de Farmácia Universidade do Porto Porto Portugal; ^4^ ICBAS Instituto de Ciências Biomédicas de Abel Salazar Universidade do Porto Porto Portugal

**Keywords:** acetylcholinesterase, antifouling, epitope map, STD‐NMR, tyrosinase

## Abstract

Ecofriendly and sustainable antifouling (AF) compounds are required to replace toxic additives in maritime AF coatings. Our group has developed synthetic AF compounds with anti‐settlement activity toward *Mytilus galloprovincialis* mussel with nontoxic properties against this target organism. Some compounds have shown to be capable of modulating the activity of key enzymes involved in mussel settlement, namely, tyrosinase and acetylcholinesterase (AChE). The saturation transfer difference nuclear magnetic resonance (STD‐NMR) technique is a powerful ligand‐based approach to disclose the moieties responsible for binding to macromolecules in solution. This work aimed to study the binding mode of two AF compounds, a xanthone and a polyphenol, with tyrosinase and AChE, respectively, by using STD‐NMR. The obtained results showed that the tyrosinase inhibitor exhibited an epitope map based on the hydroxylated aromatic ring, whereas the AChE inhibitor established interactions with both the aromatic ring and the aliphatic moiety. Further competition assays with established inhibitors, namely, kojic acid for tyrosinase and eserine for AChE, suggested that the xanthone derivative engages tyrosinase in a competitive manner, whereas the polyphenol interacts with AChE at sites distinct from the catalytic active site. These structural insights will help the rational design for optimized AF agents by targeting tyrosinase and AChE.

## Graphical abstract

Mussels are key macrofoulers targeted by antifouling (AF) strategies, yet few molecular targets have been explored to guide the development of new ecofriendly agents. Here, saturation transfer difference‐nuclear magnetic resonance (STD‐NMR) is used to investigate the interactions between AF compounds and settlement‐related enzymes, namely, tyrosinase and acetylcholinesterase, with the aim of identifying pharmacophoric features to advance AF discovery.

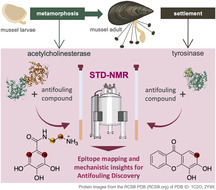

## 1 Introduction

**FIGURE 1:**
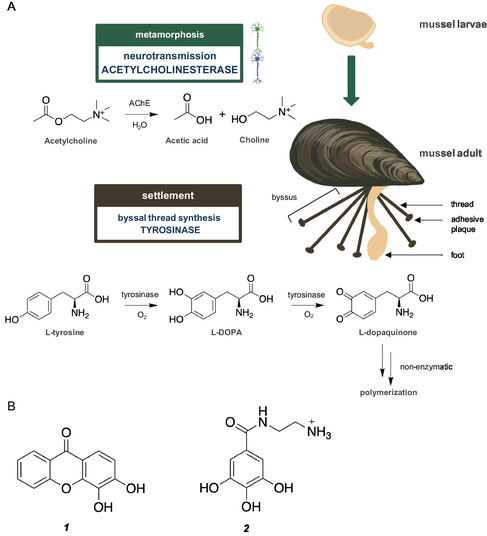
(A) AChE and tyrosinase enzymes enrollment in mussel metamorphosis and settlement. (B) Structures of nature‐inspired synthesized compounds investigated herein with reported anti‐settlement activity against mussel's larvae and with inhibitory enzymatic activity on tyrosinase (**1**) or AChE (**2**). AChE: acetylcholinesterase; L‐DOPA: levodopa.

Marine biofouling is the natural colonization of submerged materials by micro and macroorganisms having a significant impact on the marine industry, with higher fuel consumption and maintenance costs, and on ecosystems by transporting species with invading potential to new destinations [1]. Antifouling (AF) coatings are effective strategies to overcome this natural process; however, they rely on copper and booster biocides, which present hazards to marine life [2]. Therefore, under the scope of the United Nations Ocean Decade, new and ecofriendly AF agents are demanded by the international community. Synthetic AF compounds with nontoxic profile were previously reported by our group [3, 4, 5, 6, 7, 8, 9, 10]. Some of these compounds seemed to slightly modulate the activity of acetylcholinesterase (AChE) and tyrosinase [3, 4, 7, 9, 10, 11], which are involved in neurological functions and in byssal thread's biosynthesis, respectively, in macrofoulers like mussels (Figure 1A) [12].

Tyrosinase is the key enzyme initiating the biosynthetic pathway of melanin, playing a crucial role in different organisms (animals, plants, fungi, and bacteria). It is a phenoloxidase that catalyzes the *o*‐hydroxylation of phenol (L‐tyrosine) to catechol (L‐DOPA) and its oxidation to quinone (L‐dopaquinone) (Figure 1A), which occurs with the parallel reduction of molecular oxygen to water [13]. This enzymatic transformation is relevant in mussels, as the L‐dopaquinone form is the building block of byssal threads, which are responsible for adhesion to the substrate and settlement of the animal [14]. Some AF compounds, namely, hemibastadin derivatives [15, 16, 17], and *N*‐substituted maleimides and succinamides derivatives [18], were described as inhibitors of the blue mussel phenoloxidase.

Another enzyme that is also identified as an AF target is AChE, an enzyme involved in neurotransmission, by degrading the available acetylcholine, which plays an important role in mussel larvae metamorphosis and foot exploratory activity (Figure 1A) [19, 20, 21]. By inhibiting AChE, neurotoxicity can be reached due to excess of acetylcholine by hampering the turnover of this neurotransmitter. The AF biocide Sea‐Nine (4,5‐dichloro‐2‐*n*‐octyl‐4‐isothiazolin‐3‐one; DCOIT), widely used due to its broad‐spectrum activity against major fouling organisms, is well known for its inhibitory activity of AChE [22]. Also, a derivative of benzimidazolone was shown to be a potent cholinesterase inhibitor of electric eel AChE and a potent inhibitor of larval metamorphosis in the sea squirt *Ciona savignyi* [23].

Saturation transfer difference nuclear magnetic resonance (STD‐NMR) is a popular ligand‐based methodology with many applications in drug discovery and chemical biology. It works by selectively saturating the macromolecule's proton signals; if a ligand binds, magnetization is transferred from the protein to the nearby protons of the ligand. When the ligand dissociates, this transferred saturation persists, resulting in a reduction in the intensity of its proton signals—the basis for detecting binding [24, 25]. Therefore, STD‐NMR has been contributing to the characterization of molecular interactions of small molecules (with intermediate to low affinities) with target macromolecules in solution, mapping the epitope in the small molecule, a valuable information to proceed with hit/lead optimization and validate in silico predictions [26].

To support the rational design of optimized AF agents targeting tyrosinase and AChE, this work applied for the first time the STD‐NMR technique in the AF field to investigate protein‐ligand interactions of two patented compounds with AF activity against the settlement of *Mytilus galloprovincialis* larvae, namely, a xanthone derivative (**1**) and a polyphenol derivative (**2**) (Figure 1B) [3, 7, 11, 27, 28]. The results confirmed the molecular binding of these AF compounds to mushroom tyrosinase and electric eel AChE and provided structural information to construct binding epitope maps identifying the ligand moieties involved in enzyme recognition. Furthermore, competition assays with established inhibitors enabled discrimination between competitive and noncompetitive binding behaviors of the AF compounds. Altogether, these findings demonstrate the value of STD‐NMR as a mechanistic tool to elucidate enzyme recognition relevant to AF discovery.

## 2 Results and Discussion

The application of NMR methodologies in marine chemical ecology spans metabolite's structure elucidation, dereplication, and metabolomics of marine organisms [29, 30]. Within this landscape, STD‐NMR provides experiment‐first evidence of intermolecular interactions in solution, namely, bioactive candidates binding to target macromolecules, and identifies which ligand moieties contact that macromolecule under aqueous conditions [31]. Unlike docking and molecular dynamics, which deliver hypothetical poses, STD‐NMR reports binding, epitope patterns, affinity determination, and through competition, spatial information like binding site overlap [32, 33]. In combination with WaterLOGSY, it enables sensitive detection of weak binders and deconvolution in mixtures, streamlining hit screening from complex sources, namely, marine‐based extracts [34]. However, these attributes have been widely exploited in chemical biology and natural products research, their application to AF targets has been largely unexplored. In this work, we explore the application of STD‐NMR in the AF field for the first time.

For a successful STD‐NMR experiment, the ligand must be in excess, with protein:ligand ratio typically ranging from 1:50 to 1:1000. Therefore, limited solubility of bioactive compounds in protein aqueous buffers can pose a significant challenge [25]. As such, among our library of nature‐inspired synthesized compounds with reported AF activity in mussels *M. galloprovincialis*, only those exhibiting inhibitory activity against mushroom tyrosinase or electric eel AChE and adequate solubility in aqueous solution—compounds **1** and **2** (Figure 1B)—were selected for STD‐NMR studies. Despite interspecies sequence divergence, the catalytic architecture and substrate‐recognition motifs of both tyrosinase and AChE are highly conserved across taxa [35, 36, 37, 38]. The on‐ and off‐resonance irradiation frequencies were selected based on the ^1^H‐NMR spectra of the enzymes and compounds. Optimal on‐resonance frequencies were identified at 2.03 ppm for tyrosinase and 1.68 ppm for AChE, corresponding to regions rich in protein aliphatic resonances, and the off‐resonance was set at 60 ppm. Additionally, competition assays were performed with known inhibitors of tyrosinase or AChE, namely, kojic acid (KA) and eserine (ESE), respectively, to further characterize the binding mode of these AF compounds. Of note that, the %STD values reported herein are semiquantitative and based on a single saturation time. Although differences in T_1_ relaxation can affect relative STD intensities, the results are consistent with the expected binding regions and are interpreted assuming minimal T_1_ variation among ligand protons [39]. The calculated STD amplification factor (STD‐AF) and %STD for each STD signal are summarized in Tables S1‐S2 in Supporting Information.

### 2.1 Antifouling Compound 1 and Tyrosinase

**FIGURE 2:**
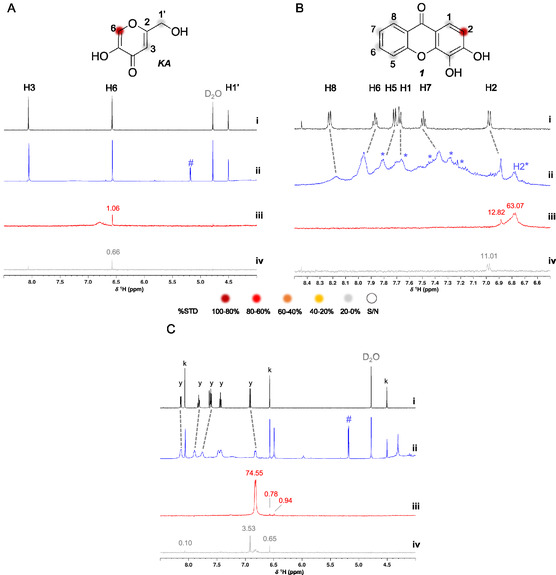
AF compound 1 competes with KA for the active site of tyrosinase. (A–C) STD‐NMR analysis of (A) tyrosinase + KA, (B) tyrosinase + compound **1**, and (C) tyrosinase + KA + compound **1** (competition assay). (i ‐ black) Reference ^1^H NMR spectra of KA, compound **1,** and KA + compound **1**, each at 1000 µM, recorded in buffered D_2_O/DMSO‐d_6_ (95:5, pH 7.2). (ii ‐ blue) Off‐resonance spectra, and (iii ‐ red) STD spectra of the enzyme‐ligand(s) mixtures (tyrosinase:ligand = 1:100). (iv ‐ gray) Control STD spectra of the free ligands, recorded under identical conditions. On‐resonance = 2.03 ppm; off‐resonance = 60 ppm. Dashed gray lines indicate chemical‐shift changes upon binding. The symbol (*) marks signals from the quinone form of compound **1**, and (#) denotes the anomeric signal of the tyrosinase sample. STD amplification factor values are shown above the corresponding ligand resonances. Color scale for %STD: 100%–80% dark red, 80%–60% red, 60%–40% orange, 40%–20% yellow, and < 20% gray (no STD). Labels: k = protons of kojic acid; y = protons of compound **1**.

To establish the STD‐NMR experimental conditions to study the protein–ligand interaction of AF compound **1** with mushroom tyrosinase, the binary tyrosinase/KA was used as a reference for tyrosinase inhibition (Figure 2A), as described by Pires and colleagues (2021) [40]. The ^1^H‐NMR spectrum of tyrosinase alone showed a sharp proton signal at δ_H_ 5.2 ppm (Figure S1, Supporting Information), characteristic of anomeric protons [41]. This observed resonance might be derived from carbohydrate residues arising from the mushroom tyrosinase sample. The ^1^H NMR spectrum of KA alone showed three proton signals, two aromatic (δ_H_ 8.08 and 6.58 ppm) and one aliphatic at a relatively downfield chemical shift (δ_H_ 4.51 ppm) due to its proximity to a primary alcohol and to the aromatic ring (Figure 2A, black). Neither the phenolic nor the alcoholic protons gave observable signals, attributable to exchange with deuterium from the solvent [D_2_O/DMSO‐d_6_ (95:5)] [42]. For the STD experiment, KA was used at 1000 µM, with an enzyme:ligand ratio of 1:100, in 5% DMSO‐d_6_ and buffered D_2_O, and the STD experiment was performed by applying 2.03 ppm (on‐resonance) and 60 ppm (off‐resonance) selective pulses. The STD spectra evidenced that the aromatic proton H‐6, vicinal to the phenolic moiety, received saturation from the protein (Figure 2A, red). Afterward, those conditions were applied to STD‐NMR experiments with tyrosinase/compound **1** (Figure 2B). The ^1^H‐NMR spectrum of compound **1** alone showed a total of six aromatic proton signals between δ_H_ 9–6 ppm (Figure 2B, black), without a trace of the phenolic protons, due to the exchangeable nature of these protons with deuterium from the solvent [D_2_O/DMSO‐d_6_ (95:5)] [42]. In the presence of tyrosinase, all proton signals shifted: most resonated at lower ppm, whereas H‐5 and H‐6 resonated at higher ppm, indicating increased deshielding. The signals also showed loss of multiplicity, broadening and the appearance of new signals, namely, H‐2* (δ_H_ 6.90 ppm), and signals (*) overlapping with H‐5 (δ_H_ 7.90–7.77 ppm), H‐1 (δ_H_ 7.70–7.61 ppm), and H‐7 (δ_H_ 7.50–7.23 ppm) (Figure 2B, blue). These observations already indicated a binding event. Such alterations are consistent with changes in molecular tumbling and relaxation times upon complex formation, which typically result in line broadening and slight shift perturbations in the bound state [26, 43]. Importantly, the mixture and free compounds were exactly in the same conditions (temperature, concentration, solvent, and pH), ruling out experimental conditions as the source of the observed spectral changes. Also, compound **1**, being a catechol, can be partially oxidized by tyrosinase to its corresponding quinone. As a result, both redox forms may coexist in equilibrium and generate additional proton signals (*) with distinct chemical shifts. The quinone form, in particular, produced a more shielded H‐2*, consistent with MNova predictions and with the spectrum obtained after potassium dichromate‐based oxidation (Figure S2, Supporting Information). The broader quinone resonances likely reflect a combination of paramagnetic relaxation enhancement (PRE) arising from transient semiquinone/Cu(II) states generated during tyrosinase turnover and intermediate‐timescale exchange within the catechol‐quinone redox manifold in aqueous solution [44, 45, 46]. Also, the broader lines in inorganic oxidation of compound **1** could be related with Cr(III) from dichromate (Figure S2, Supporting Information) [47]. A shift of the vicinal aromatic proton of a quinone toward a more shielded region was also reported by Ando and colleagues (2010) [48]. For compound **1**, the STD was observed for only one proton, the H‐2, nevertheless, another more shielded STD signal was detected with higher relative intensity, corresponding to the proton H‐2* of the quinone form (Figure 2B, red, and Figure S2, Supporting Information). Therefore, compound **1** exhibited STD signals, validating the binding of compound **1** to tyrosinase, in agreement with the previously observed inhibition of enzymatic activity [11]. The epitope mapping of compound **1** evidenced that the hydroxylated aromatic ring is responsible for the interaction with tyrosinase.

**FIGURE 3:**
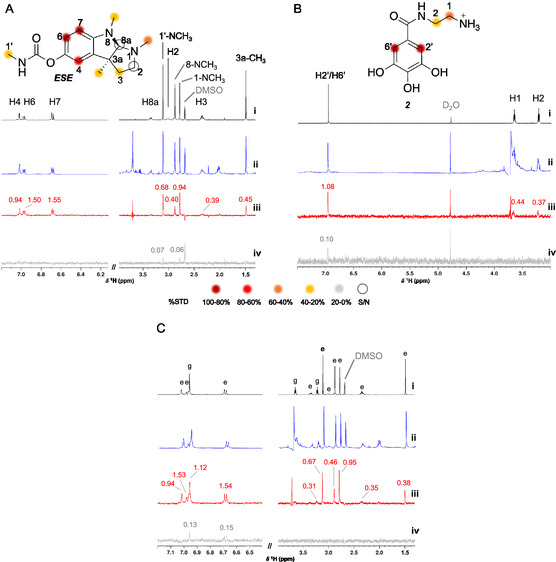
AF compound 2 does not compete with ESE for the active site of AChE. (A–C) STD‐NMR analysis of (A) AChE + ESE, (B) AChE + compound **2**, and (C) AChE + ESE + compound **2** (competition assay). (i – black) Reference ^1^H NMR spectra of ESE, compound **2**, and ESE + compound **2**, each at 1000 µM, recorded in buffered D_2_O/DMSO‐d_6_ (95:5, pH 7.2). (ii – blue) Off‐resonance spectra, and (iii – red) STD spectra of the enzyme‐ligand(s) mixtures (AChE:ligand = 1:100). (iv – gray) Control STD spectra of the free ligands, recorded under identical conditions in the absence of enzyme. On‐resonance = 1.68 ppm; off‐resonance = 60 ppm. STD amplification factor values are shown above the corresponding ligand resonances. Color scale for %STD: 100%–80% dark red, 80%–60% red, 60%–40% orange, 40%–20% yellow, and < 20% gray (no STD). Labels: e = protons of ESE; g = protons of compound **2**.

A tyrosinase competition experiment was performed with KA and compound **1**, at identical conditions as the binary mixtures. In the ternary mixture (KA and compound **1** with tyrosinase), the off‐resonance spectrum differed from the binaries (Figure 2, blue spectra). Indeed, compound **1** lines became more intense and less broadened (Figure 2C, blue), suggesting a shift toward faster exchange and/or reduced paramagnetic broadening (*e.g.*, weaker exposure to Cu(II)/semiquinone PRE when KA is present near the dicopper center of tyrosinase), consistent with competition at or near the catalytic site [43, 44, 45]. Also, in the ternary mixture, the aromatic proton H‐7 of compound **1** did not shift, as observed in the binary mixture, suggesting a different binding mode. Notably, in the presence of KA, the second line for H‐2* resonance assigned to the quinone form of compound **1** was no longer detected (Figure 2C, blue). This is consistent with KA inhibiting tyrosinase turnover, thereby suppressing the catechol‐quinone conversion of compound **1** and eliminating the bound quinone microstate that generated the H‐2* STD signal in the binary mixture. In its turn, KA showed duplication of H‐6, indicative of two slowly exchanging microstates (Figure 2C, blue) [43]. These two lines are consistent with KA adopting two distinct bound geometries—one likely involving Cu(II) coordination and another stabilized by hydrogen‐bonding or peripheral interactions [49, 50]—whose slow interconversion on the NMR timescale results in two separate H‐6 resonances visible in the STD spectrum (Figure 2C, blue). Accordingly, when comparing the STD spectra of the binary mixtures with that of the ternary one, it was observed that the intensity of the signal corresponding to H‐2 of compound **1** increased, while that of H‐6 of KA decreased (Figure 2, red spectra). This apparent enhancement of the H‐2 signal intensity reflects a shift in bound‐state dynamics. Because KA inhibits catalytic turnover, compound **1** remains predominantly in catechol form and exchanges more rapidly between free and bound states, which increases STD efficiency [26, 43, 51]. In contrast, the H‐6 signal intensity of KA decreased in the ternary mixture probably due to reduced occupancy of the catalytic site.

Structurally, tyrosinase is a copper enzyme that possesses two superficial allosteric sites (1 and 2) and one catalytic site containing two nonidentical copper ions, each coordinated by three histidine residues (His‐61, His‐85, His‐94, and His‐259, His‐263, His‐296) [35, 52]. Tyrosinase's cresolase and catecholase activities depend on catalytic turnover involving enzymatic intermediates [37]. KA, a known moderate tyrosinase inhibitor (µM range), is considered a transition‐state analog, as it shares structural features with catechol (the natural substrate), and its activity depends on catalytic turnover but it cannot undergo oxidation [53, 54]. It was demonstrated that KA had a higher affinity to the *deoxy* and *oxy* forms of tyrosinase during catalytic turnover [53, 55]. A theoretical study of tyrosinase inhibitor binding kinetics showed that KA was predominantly competitive for the *deoxy* form, whereas it was mainly noncompetitive when the substrate had a greater affinity to the *oxy* form [55]. Furthermore, KA was described to bind to both catalytic and allosteric site 2, being considered a mixed‐type inhibitor [50]. Bochot and colleagues (2014) disclosed the binding mode of KA to bacterial tyrosinase using a dicopper complex by crystallographic and in silico methodologies, showing that the KA oxygen atoms (enol moiety) were involved in a bidentate coordination with the two copper ions of tyrosinase active site [49]. Additionally, Deri and colleagues (2016) elucidated the coordination of KA oxygens with the bacterial binuclear copper‐binding site that occurred via a water molecule and the importance of the active site's histidines in the metal‐coordination and *π*–*π* stacking interactions with the kojic aromatic ring [52]. Pires and colleagues (2021) demonstrated the interaction of KA and a new class of tyrosinase inhibitors, namely, isobenzonfuran‐1‐(3*H*)‐ones, with tyrosinase by STD‐NMR, highlighting the involvement of aromatic protons in the interaction and suggesting additional contacts with different residues within the enzyme's binding cavity [40]. In the present work, it was also evidenced that the aromatic proton H‐6 was involved in KA interaction with tyrosinase; however, the aromatic proton H‐3 did not showed STD signal (Figure 2A, red), indicating that proton H‐6 was in a more accessible or spatially favored position to receive tyrosinase's saturation.

In compound **1**, the aromatic proton H‐2 gave a clear STD signal (Figure 2B, red), whereas the vicinal phenolic protons (3‐OH and 4‐OH) were not detected, likely due to their rapid exchange with deuterium from the solvent [42]. Notably, the results also showed that compound **1** can be oxidized to its quinone form, as evidenced by the appearance of new proton resonances, including H‐2*, which likewise exhibited an STD response. These observations suggest that ligand recognition may involve the catechol moiety, which is structurally analogous to the natural substrate accommodated within the tyrosinase active site [13]. Moreover, in a previous study, compound **1** displayed docking scores comparable to those of KA at both the active site and allosteric site 2 of tyrosinase [11], supporting the possibility that compound **1** could act as a mixed‐type inhibitor. The absence of STD signal for H‐1, despite the strong signal for the adjacent H‐2/H‐2*, reflects the spatial nature of saturation transfer in STD‐NMR. H‐2 is likely positioned closer to the binding site, whereas H‐1 remains solvent‐exposed or has a less favorable orientation, resulting in negligible saturation transfer. Similar orientation‐dependent selectivity among neighboring aromatic protons has been observed in other protein‐ligand complexes and in L‐DOPA docking to tyrosinase [56, 57]. Interestingly, quinone proton H‐2* exhibited a higher relative STD signal than the catechol H‐2, because of a more efficient interaction with tyrosinase. This observation hints about a stronger binding of the quinone form of compound **1** to tyrosinase, consistent with a higher apparent affinity or a more stable binding mode or, alternatively, it could also reflect a faster exchange or turnover rate between free and bound states [58]. These findings suggest that both redox forms may interact with tyrosinase, but the quinone is likely the most efficiently recognized and thus the predominant inhibitory species under oxidative conditions. The competition data support a competitive binding scenario in which KA prevents the conversion of compound **1** into its quinone form by occupying the catalytic site, while compound **1** may additionally interact with one or both allosteric sites, as suggested by the persistence of the H‐2 STD signal of the cathecol form even when catalytic turnover is blocked. In a mixed‐type scenario, compound **1** would bind to the catalytic site whereas KA would bind to allosteric site 2, inhibiting tyrosinase activity. Further characterization of the catechol‐quinone equilibrium and the relative affinities of each redox form for the catalytic and allosteric sites would help to refine this mechanistic model.

### 2.2 Antifouling Compound 2 and AChE

To establish the STD‐NMR experimental conditions to study the interaction of compound **2** with electric eel AChE [7], the binary AChE/ESE was used as a reference for AChE interaction (Figure 3A), with the conditions protein:ligand ratio of 1:100 (ESE at 1000 µM) in 5% DMSO‐d_6_, in buffered D_2_O, using 1.68 ppm (on‐resonance) and 60 ppm (off‐resonance) as selective pulses. Afterward, those conditions were applied to STD‐NMR experiment of AChE/compound **2** (Figure 3B). The ^1^H‐NMR spectrum of ESE alone exhibited a total of three aromatic proton signals (δ_H_ 7.1–6.6 ppm) and seven aliphatic proton signals (δ_H_ 3.4–1.4 ppm) (Figure 3A, black). The STD experiment evidenced that ESE binds to AChE, showing that the aromatic protons (H‐4, H‐6, and H‐7) received saturation from the protein in a higher percentage, the aliphatic protons from methyl substituents (1'‐NCH_3_, 1‐NCH_3_, 8‐NCH_3_, and 3a‐CH_3_) also exhibited STD signals with considerable intensity; however, it was not possible to integrate the signal of H‐8a and H‐2 as their signals have a very low signal:noise ratio (Figure 3A, red).

The ^1^H‐NMR spectrum of compound **2** alone showed one aromatic proton signal, which integrated to two protons (H‐2' and H‐6'), at δ_H_ 6.96 ppm, and four aliphatic proton signals (H‐1 and H‐2), between δ_H_ 4–3 ppm (Figure 3B, black). The phenolic and amine protons were not visible due to the exchange of the protons with the deuterated solvents used [42]. Compound **2** in the presence of AChE showed that the signals of protons H‐1 and H‐2 lost their triplet shape and were slightly broadened when compared to the spectrum of the compound alone, indicating a binding event, and H‐1 signal overlapped with the protein's signals (Figure 3B, blue). Upon selective irradiation, it was possible to detect STD signals with higher intensity for the aromatic protons H‐2' and H‐6' and aliphatic proton H‐1, followed by aliphatic proton H‐2 (Figure 3B, red). Therefore, based on the epitope mapping, both aromatic and aliphatic protons of compound **2** are involved in AChE binding.

A competition STD‐NMR experiment was conducted with ESE and compound **2**, at identical conditions as in the binary mixtures. In the ternary mixture (ESE and compound **2** with AChE), the off‐resonance spectrum showed no appreciable changes relative to the binary mixtures: both compounds retained similar proton line widths and chemical shifts (Figure 3C, blue). Consistently, the STD spectrum of the ternary mixture displayed comparable STD‐AF values to those obtained in the individual binary experiments, indicating no significant change in bound fraction or exchange regime upon co‐incubation (Figure 3C, red). These results suggest that compound **2** does not compete for the catalytic gorge of AChE, where ESE binds reversibly [59], but rather interacts with a distinct, possibly peripheral or allosteric region of the enzyme. Altogether, the absence of STD‐AF attenuation for either ligand in the ternary mixture supports non‐overlapping binding sites and the coexistence of ESE and compound **2** binding to AChE under the tested conditions.

AChE is a serine hydrolase and its active site is a narrow, long and hydrophobic cavity that is divided in two subsites: the esteratic subsite, where the catalytic amino acids Ser‐203, His‐447, and Glu‐334 are at the bottom of the gorge where acetylcholine is hydrolyzed into acetate and choline [60]; and the anionic subsite, located in the entrance of the catalytic site, composed by Trp‐86, Glu‐202, and Tyr‐337, where the quaternary trimethylammonium group of acetylcholine binds [61]; which are connected by the gorge, a hydrophobic narrow region, composed by aromatic residues (Tyr‐72, Asp‐74, Tyr‐124, Glu‐285, Trp‐286, and Tyr‐341), that traps acetylcholine and transfers it to the deep catalytic site [60, 62]. Some AChE inhibitors have been described to bind to the catalytic site, namely, ESE, a pseudo‐irreversible inhibitor. It first binds reversibly at the esteratic subsite and subsequently carbamylates the active‐site serine, effectively blocking the gorge and preventing access to the substrate [59, 63]. Compound **2** exhibited STD signals for all its aromatic and aliphatic protons (Figure 3B). These results are aligned with the STD epitope map obtained for ESE (Figure 3A), as well with other STD‐NMR studies on galantamine [64], 4‐methylumbelliferone [65], an hydroxycoumarin, and rosmarinic acid [66] with AChE inhibitory activity, showing that the entire molecules were involved in binding to electric eel AChE. Other STD‐NMR study with dicationic cholinium ionic liquids, structurally analogous to acetylcholine, demonstrated that these molecules occupy the hydrophobic narrow region of AChE and competitively displace galantamine [67]. In another report, a ruthenium–II complex bearing a 4,2‐ethylamino‐pyridine substituent exhibited increased binding efficiency to human AChE, further supporting the influence of cationic side chains on binding orientation [68]. Given the structural similarity of compound **2** to acetylcholine, particularly the side chain containing an amide group separated by two methylene units from a protonated amine, the interaction was expected to occur within the catalytic gorge of AChE. However, the competition assay with ESE revealed that compound **2** binds instead to peripheral or allosteric regions of the enzyme, consistent with a noncompetitive inhibitory profile. Compound **2** is a derivative of the polyphenol gallic acid, which has been described as a competitive inhibitor of AChE, similarly to galantamine. STD‐NMR studies have shown that the aromatic protons of gallic acid are directly involved in strong binding that cannot be displaced by coumarin‐derived inhibitors [65]. In contrast, our results suggest that the structural modification in compound **2**, namely, the introduction of an amide‐amine side chain, alters the binding topology and redirects the interaction away from the active site. Notably, rosmarinic acid, a polyphenol, was reported not to compete with the positively charged inhibitor donepezil, indicating binding at a site distinct from the deep catalytic gorge of AChE [66]. Taken together, these findings suggest that small structural variations in cholinergic scaffolds can significantly influence binding site preference and the inhibition mechanism. Future enzymatic activity‐based competition assays would be valuable for further characterizing the noncompetitive binding mode of compound **2**, as suggested by the present STD‐NMR data.

## 3 Conclusion

For tyrosinase binding, the hydroxylated aromatic scaffold of compound **1** showed to be crucial for binding, suggesting that metal coordination and *π*–*π* stacking occur. The catechol moiety of compound **1** acts as a substrate of tyrosinase, as evidenced by the appearance of new signals indicative of quinone formation, suggesting that the molecule binds to the active site, confirmed by the competition assay in the presence of KA. The putative binding pockets of tyrosinase seem to be wider or more superficial, as the number of interactions is more limited when compared with the number of STD signals obtained with ESE and compound **2** with AChE. Indeed, for AChE binding, all protons of compound **2** exhibited STD, showing that the aromatic and aliphatic protons were required for binding, probably involving polar and hydrophobic interactions, like hydrogen bonds and *π*–*π* stacking. The competition assay of compound **2** showed that it does not compete with ESE to the catalytic site of AChE, suggesting that compound **2** binds to an allosteric site.

Although STD‐NMR has been widely used in natural products and chemical biology studies, our work brings this mature tool from chemical ecology into the AF field, reporting direct binding and epitope patterns that complement phenotypic assays with target organisms, enzymatic activity‐based screenings, and in silico pipelines. Overall, the robustness and sensitivity of STD‐NMR for binding detection, epitope mapping, and the inference on the influence of the protein chemical environments, prove the informative capacity of this ligand‐based biophysical technique on binding modes for tyrosinase and AChE. Together with previous structure–activity relationships, in silico docking and, in vitro enzymatic assays, the present STD‐NMR data contributes to the construction of a pharmacophore model for AF compounds targeting tyrosinase or AChE. This is a crucial step toward the rational design of optimized AF agents.

## 4 Experimental Section

### 4.1 General

All chemicals and reagents were purchased from Sigma–Aldrich (Missouri, USA). Regular and deuterated solvents were analytical grade. Enzymes were purchased from Sigma–Aldrich and used without further purification processes: mushroom (*Agaricus bisporus*) tyrosinase (T3824, 6540 U/mg) and electric eel (*Electrophorus electricus*) AChE (C3389, 253 U/mg). Commercial inhibitors were purchased: kojic acid (KA) (K0010; Tokyo Chemical Industry Co., Ltd. (TCI), Tokyo, Japan) and eserine (ESE) (E8375; Sigma–Aldrich). Compounds 3,4‐dihydroxy‐9*H*‐xanthen‐9‐one (**1**) [3] and 2‐(3,4,5‐trihydroxybenzamido)ethan‐1‐aminium hydrochloride (**2**) [7] were selected from our library of AF compounds.

### 4.2 Sample Preparation

Three sets of 5 mm NMR tubes were prepared: i) free compounds **1** and **2**, and reference inhibitors (KA and ESE) (control experiments), ii) free enzymes, and iii) mixture of enzyme/compound(s). Stock solutions of all compounds were prepared in DMSO‐d_6_ at 60 mM, whereas enzyme's stock solutions were prepared in D_2_O at 60 µM, and stored at −20°C.

#### 4.2.1 Binding Assays

For the binding assays, KA, ESE, and compounds **1** and **2** alone were diluted in 10 mM phosphate buffer (pH 7.2) in D_2_O/ DMSO‐d_6_ (95:5) at a final concentration of 1000 µM. Tyrosinase and AChE alone were diluted in the same buffered solvent at a final concentration of 10 µM. Mixtures of enzyme/ligand were prepared with molar ratios of 1:100 (10 μM of enzyme:1000 μM of ligand) in the same buffered solvent, resulting in a final volume of 600 µL.

#### 4.2.2 Competition Assays

For the competition assays, the control, a mixture of testing compound (**1** or **2;** 1000 µM) and reference inhibitor (KA or ESE; 1000 µM) was prepared in 10 mM phosphate buffer (pH 7.2) in D_2_O/ DMSO‐d_6_ (95:5). For the competition experiment, in the same tube was added the enzyme (10 µM), the testing compound and the reference inhibitor (each at 1000 µM, molar ratio of enzyme/ligand 1:100), in 10 mM phosphate buffer (pH 7.2) in D_2_O/ DMSO‐d_6_ (95:5), resulting in a final volume of 600 µL.

### 4.3 NMR Spectroscopy

#### 4.3.1 ^1^H‐ and STD‐NMR Experiments

^1^H‐ and STD‐NMR experiments were recorded at CEMUP‐Materials Centre of the University of Porto on a Bruker Avance III HD 600 MHz spectrometer equipped with a 5‐mm prodigy cryogenic probe head at 298 K. ^1^H‐NMR characterization of the free compounds **1** and **2**, KA, and ESE, as well as the enzymes (tyrosinase and AChE), was performed in the same concentrations and solvent system used for STD‐NMR analysis. This information allowed us to decide the frequencies for pulse irradiation on the macromolecule, without targeting compound's signals. ^1^H‐NMR spectra of the mixtures were also recorded. ^1^H‐NMR spectra were acquired with 32–64 scans for compounds and 80 scans for enzymes, using water suppression via excitation sculpting with gradients, in a spectral window of 9,615.38 Hz centered at 2,868.39 Hz.

STD‐NMR experiments were recorded with a spectral width of 9,615.38 Hz centered at 2,868.24 Hz with water suppression with 2 ms shaped pulse. The selective irradiation was obtained by the application of a series of soft Gaussian‐shaped pulses with 50 ms of length, with the best saturation time at 2.5 s, relaxation time (D1) of 3.5 s, and 20 cycles of number averages (L4).

Selective irradiation (saturation) of tyrosinase was at 1,215.72 Hz (2.03 ppm) for on‐resonance and 36,000 Hz (60 ppm) for off‐resonance frequencies, whereas for the experiments with AChE, the selective irradiation was performed at 1,007.44 Hz (1.68 ppm) for on‐resonance and 36,000 Hz (60 ppm) for off‐resonance. A total of 120 scans for compounds **1** and for KA in the presence of tyrosinase, and 48 scans for compounds **2** and ESE in the presence of AChE were acquired.

Bruker TopSpin 3.6.2 software was used for acquisition and version 4.3.0 for processing (Bruker, Billerica, Massachusetts, USA). Control STD experiments of the free compounds were carried out, under the exact same experimental conditions of the mixtures (temperature, concentration, solvent, and pH), to understand if the free compounds would receive any direct saturation by the chosen frequency pulses [67]. STD effects were quantified by integrating identical proton regions in the off‐resonance and STD spectra; the STD amplification factor (STD‐AF) was calculated accordingly with Equation (1), and the corrected ΔSTD‐AF with Equation (2). The percentage of STD (%STD) for epitope mapping was determined with Equation (3), by normalizing to the most intense ligand proton (100%).

(1) $$\mathrm{STD}\mathrm{AF}=\left(\frac{I_{\mathrm{STD}}}{I_{\mathrm{off}}}\right)\times \left(\frac{\left[L\right]}{\left[E\right]}\right)$$

$$I_{\mathrm{STD}}=\text{intensity}\;\mathrm{of}\;\mathrm{the}\;\text{proton signal}\;\mathrm{in}\;\mathrm{the}\;\mathrm{STD}\;\text{spectrum}$$

$$I_{\mathrm{off}}=\text{intensity}\;\mathrm{of}\;\mathrm{the}\;\text{proton signal}\;\mathrm{in}\;\mathrm{the}\;\text{reference}\;\mathrm{off}\;-\text{resonance spectrum}$$

[L]=ligand concentration

[E]=enzyme concentration

(2) $$\Delta \mathrm{STD}\mathrm{AF}=\left(\mathrm{STD}{\mathrm{AF}}_{\mathrm{Ligand}+\mathrm{Protein}}-\mathrm{STD}{\mathrm{AF}}_{\mathrm{Free}\mathrm{ligand}}\right)$$

(3) $$\%\mathrm{STD}=\left(\frac{\mathrm{STD}{\mathrm{AF}}_{\mathrm{proton}}}{\mathrm{STD}{\mathrm{AF}}_{\max}}\right)\times 100$$

Epitope mapping was derived from STD intensities obtained at a single saturation time (2.5 s). Because differential longitudinal relaxation times (T_1_) of ligand protons can influence relative STD intensities, the derived epitope maps were interpreted under the assumption that T_1_ values do not vary significantly among the protons of each ligand [39].

## Supporting Information

Additional supporting information can be found online in the Supporting Information section. **Supporting** **Fig.** **S1**: ^1^H‐NMR spectra of (A) tyrosinase and (B) acetylcholinesterase at 10 µM (phosphate‐buffered D_2_O/DMSO‐d_6_(95:5), pH 7.2). **Supporting** **Fig.** **S2**: The aromatic protons’ resonances of the quinone form of compound 1 present different chemical shifts. ^1^H NMR spectra predictions of (A) compound 1 and (B) its quinone form (1*) were made using Mnova software (from Mestrelab Research, S.L.). The predictions were made in D_2_O (dark blue) and DMSO‐d_6_ (light blue) to understand the influence of these solvents on proton signals. (C) Experimental ^1^H NMR spectra (600 MHz) of compound 1 (green) and its quinone form (1*; dark blue) in a mixture of D_2_O:DMSO‐d_6_ (90:10). The quinone form was obtained by oxidizing compound 1 (catechol) with potassium dichromate (K_2_Cr_2_O_7_) in a proportion of 1:1 without further purification. Dashed gray line indicate chemical shift changes. **Supporting** **Table** **S1**: Binding experiments. STD NMR results for kojic acid, eserine and compounds 1 and 2 in the presence (C+E) and absence (C) of tyrosinase or AChE. Absolute STD amplification factor (STD‐AF) values were calculated from the correspondent STD‐NMR experiments in 5% DMSO‐d_6_, with off‐resonance frequency at 60 ppm. On resonance for tyrosinase was 2.03 ppm and for AChE was 1.68 ppm. ΔSTD‐AF for each proton was calculated as the difference between the STD‐AF measured in the compound‐enzyme experiment and that measured in the control experiment. %STD for each proton was calculated by normalizing to the highest ΔSTD‐AF within the same compound. **Supporting** **Table** **S2**: Competition experiments. STD‐NMR results for the mixture of compounds alone (kojic acid + compound 1, and eserine + compound 2 – STD competition control) and in the presence of the respective enzymes (STD competition). Absolute STD amplification factor (STD‐AF) values were calculated from the correspondent STD‐NMR experiments in 5% DMSO‐d_6_, with off‐resonance frequency at 60 ppm. On resonance for tyrosinase was 2.03 ppm and for acetylcholinesterase was 1.68 ppm. D STD‐AF for each proton was calculated as the difference between the STD‐AF measured in the competition experiment and that measured in the control experiment.

## Author Contributions

**Ana Sara Gomes**: conceptualization, investigation, methodology, validation, formal analysis, visualization, writing – original draft, writing – review & editing, data curation, funding acquisition. **Mariana Andrade**: methodology, validation, writing – review & editing. **Diana I. S. P. Resende**: investigation, writing – review & editing. **Sara M. Cravo**: investigation, writing – review & editing. **Emília Sousa**: conceptualization, supervision, writing – review & editing. **Marta Correia‐da‐Silva**: conceptualization, resources, supervision, writing – review & editing, funding acquisition.

## Funding

This work was supported by the Fundação para a Ciência e a Tecnologia (UIDB/04423/2020, UIDP/04423/2020, LA/P/0101/2020, PTDC/CTA‐AMB/0853/2021, 2022.00379.CEECIND/CP1728/CT0001 and UID/04423/2025) and European Commission (C644915664‐00000026 and UID/PRR/04423/2025).

## Conflicts of Interest

M. Correia‐da‐Silva and E. Sousa are co‐inventors of the European Patent Office EP 22800324.0 (pending), 15.03.2024, “Antifouling compound, method and uses thereof”, and of the United States Patent US12030891, 09.07.2024, Chinese Patent CN113226035, 31.10.2023, Europen Patent Office EP3897150 (pending), 27.10.2021, “Xanthonic compounds and their use as antifouling agents”.

## Supporting information

Supplementary Material

## Data Availability

The data that supports the findings of this study are available in the supplementary material of this article.
