# Supplementary material for: Binding Mode Analysis of Antifouling Compounds Targeting Tyrosinase and Acetylcholinesterase by Saturation Transfer Difference NMR Spectroscopy
Source: Chembiochem. 2026 Apr 20;27(8):e70311. doi: 10.1002/cbic.70311 (PMC13096763; doi:10.1002/cbic.70311)
Supplement: Supplementary file 1 — Supplementary Material [file CBIC-27-e70311-s001.pdf]

Supporting Information

Binding Mode Analysis of Antifouling Compounds Targeting Tyrosinase and Acetylcholinesterase by Saturation Transfer Difference NMR Spectroscopy

Ana Sara Gomes*[a], Mariana Andrade[b], Diana I. S. P. Resende[a, c, d], Sara Cravo[a, c], Emília Sousa[a, c], Marta Correia-da-Silva[a, c]

[a] Ana Sara Gomes, Diana I. S. P. Resende, Sara Cravo, Emília Sousa, Marta Correia-da-Silva
CIIMAR/CIMAR LA, Centro Interdisciplinar de Investigação Marinha e Ambiental, Universidade do Porto, Terminal de Cruzeiros do Porto de Leixões, 4450-208 Matosinhos, Portugal
E-mail: sara.gomes@ciimar.up.pt

[b] Mariana Andrade
CEMUP, Centro de Materiais, Universidade do Porto
Rua do Campo Alegre, 823, 4150-180 Porto, Portugal

[c] Diana I. S. P. Resende, Sara Cravo, Emília Sousa, Marta Correia-da-Silva
Faculdade de Farmácia, Universidade do Porto
Rua Jorge de Viterbo Ferreira 228, 4050-313 Porto, Portugal

[d] Diana I. S. P. Resende
School of Medicine and Biomedical Sciences (ICBAS)
Rua de Jorge Viterbo Ferreira 228, 4050-313 Porto, Portugal

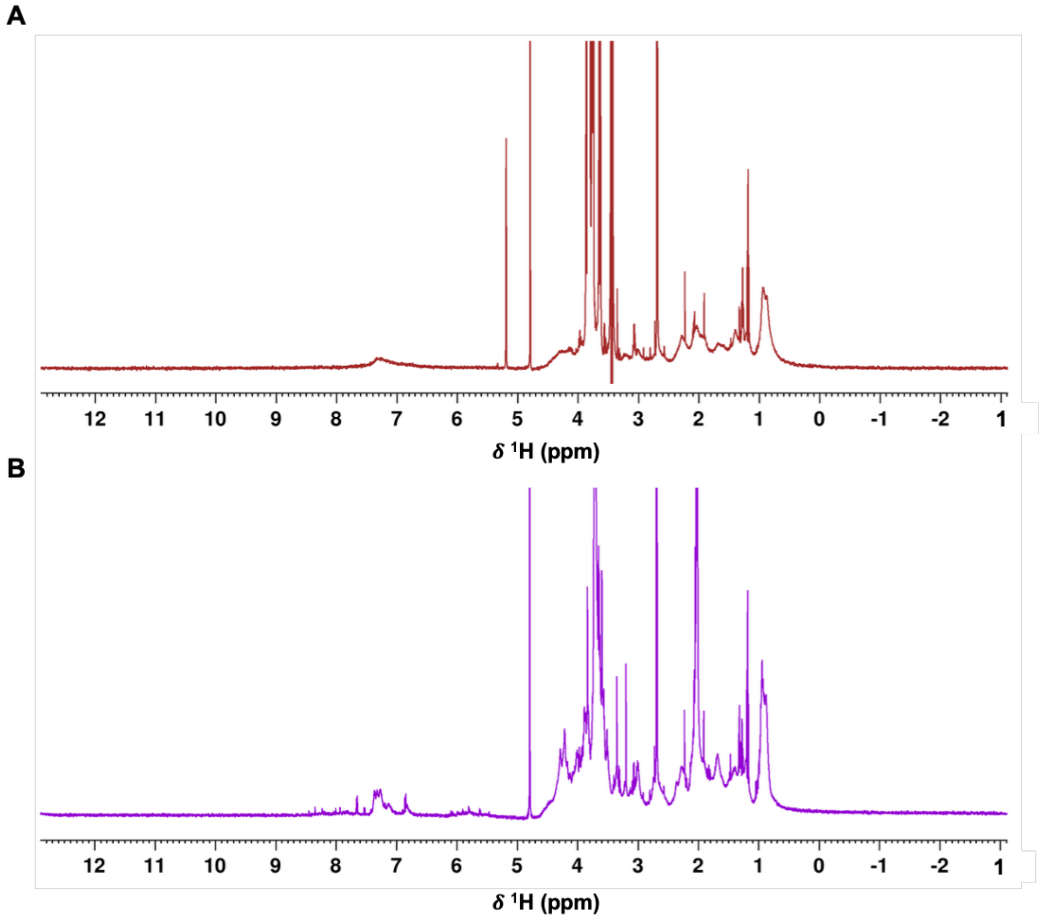

Figure S1. ^{1}H-NMR spectra of **(A)** tyrosinase and **(B)** acetylcholinesterase at 10 μM (phosphate-buffered D_2O/DMSO-d_6 (95:5), pH 7.2).

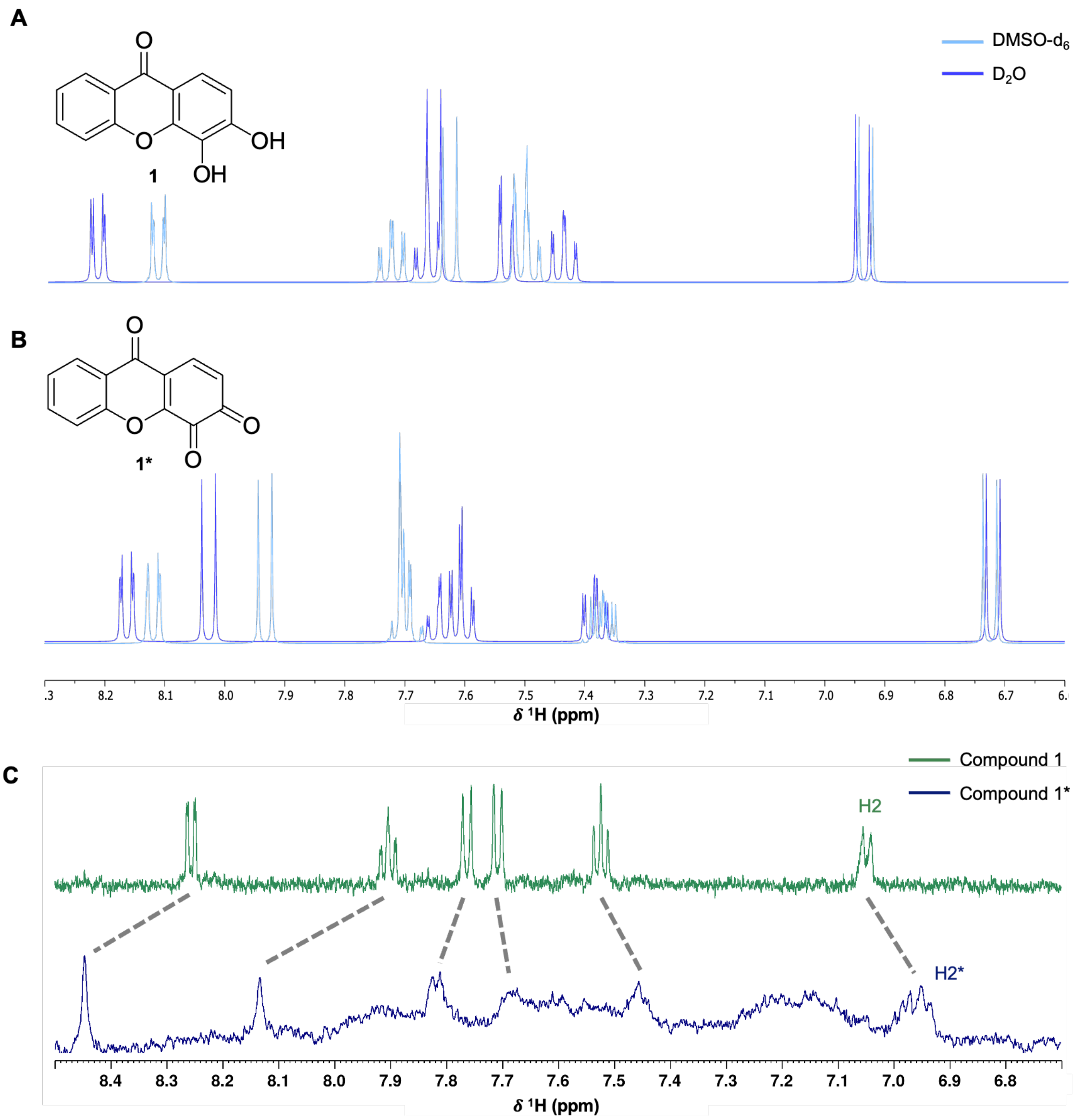

Figure S2. The aromatic protons' resonances of the quinone form of compound 1 present different chemical shifts. ^{1}H NMR spectra predictions of **(A)** compound **1** and **(B)** its quinone form (**1***) were made using Mnova software (from Mestrelab Research, S.L.). The predictions were made in D_2O (dark blue) and DMSO-d_6 (light blue) to understand the influence of these solvents on proton signals. **(C)** Experimental ^{1}H NMR spectra (600 MHz) of compound **1** (green) and its quinone form (**1***; dark blue) in a mixture of D_2O:DMSO-d_6 (90:10). The quinone form was obtained by oxidizing compound **1** (catechol) with potassium dichromate ($K_2Cr_2O_7$) in a proportion of 1:1 without further purification. Dashed gray line indicate chemical shift changes.

Table S1. Binding experiments. STD-NMR results for kojic acid, eserine and compounds **1** and **2** in the presence (C+E) and absence (C) of tyrosinase or AChE. Absolute STD amplification factor (STD-AF) values were calculated from the correspondent STD-NMR experiments in 5% DMSO-d6, with off-resonance frequency at 60 ppm. On resonance for tyrosinase was 2.03 ppm and for AChE was 1.68 ppm. Δ STD-AF for each proton was calculated as the difference between the STD-AF measured in the compound-enzyme experiment and that measured in the control experiment. %STD for each proton was calculated by normalizing to the highest Δ STD-AF within the same compound.

Compound	Proton identification	STD-AF $_{C+E}$	STD-AF $_{C\ (control)}$	Δ STD-AF	%STD (norm)
**Kojic acid**	3	S/N	0.19	No STD	-
	6	1.06	0.66	0.40	100
	1'	-	-	-	-
**1**	1	-	-	-	
	2	12.82	11.01	1.81	3
	2*	63.07	-	63.07	100
	5	-	-	-	
	6	-	-	-	
	7	-	-	-	
	8	-	-	-	
**Eserine**	1-NCH3	0.93	0.06	0.87	56
	2	S/N	-	S/N	-
	3	0.39	-	0.39	25
	3a-CH3	0.45	-	0.45	29
	4	0.94	-	0.94	61
	6	1.50	-	1.50	97
	7	1.55	-	1.55	100
	8-NCH3	0.40	S/N	0.40	26
	8a	S/N	-	S/N	-
	1'-CH3	0.68	0.07	0.61	39
**2**	1	0.44	-	0.44	45
	2	0.37	-	0.37	38
	2'	1.08	0.10	0.98	100
	6'	1.08	0.10	0.98	100

S/N – the STD signal is in the signal/noise ratio and therefore was not integrated.

Table S2. Competition experiments. STD-NMR results for the mixture of compounds alone (kojic acid + compound **1**, and eserine + compound **2** – STD competition control) and in the presence of the respective enzymes (STD competition). Absolute STD amplification factor (STD-AF) values were calculated from the correspondent STD-NMR experiments in 5% DMSO-d6, with off-resonance frequency at 60 ppm. On resonance for tyrosinase was 2.03 ppm and for acetylcholinesterase was 1.68 ppm. Δ STD-AF for each proton was calculated as the difference between the STD-AF measured in the competition experiment and that measured in the control experiment.

Compound	Proton identification	STD-AF $_{competition}$	STD-AF $_{control}$	Δ STD-AF
**Kojic acid**	3	-	0.10	No STD
	6 (a/b)	0.78/0.94	0.65	0.13/0.29
	1’	-	-	-
**1**	1	-	-	-
	2	74.55	3.53	71.02
	5	-	-	-
	6	-	-	-
	7	-	-	-
	8	-	-	-
**Eserine**	1-NCH3	S/N	-	S/N
	2	S/N	-	S/N
	3	0.35	-	0.35
	3a-CH3	0.38	-	-
	4	0.94	-	-
	6	1.53	-	-
	7	1.54	0.15	1.39
	8-NCH3	0.95	-	0.95
	8a	-	-	-
	1’-CH3	0.67	-	0.67
**2**	1	Overlapped protein	-	Overlapped protein
	2	0.31	-	0.31
	2’	1.12	0.13	0.99
	6’	1.12	0.13	0.99

S/N – the STD signal is in the signal/noise ratio and therefore was not integrated.
